# Trends and regional differences in antidiabetic medication use: a nationwide retrospective observational study

**DOI:** 10.1186/s13098-024-01334-8

**Published:** 2024-04-24

**Authors:** Márta Csatordai, Ria Benkő, Mária Matuz, Zsófia Engi, Dezső Csupor, Csaba Lengyel, Péter Doró

**Affiliations:** 1 Faculty of Pharmacy, Institute of Clinical Pharmacy, University of Szeged, Szikra utca 8, 6725 Szeged, Hungary; 2 Albert Szent-Györgyi Health Centre, Central Pharmacy, University of Szeged, Szeged, Hungary; 3 Albert Szent-Györgyi Health Centre, Department of Internal Medicine, University of Szeged, Szeged, Hungary

**Keywords:** Diabetes mellitus, Drug utilization, Antidiabetics, Regional differences, Hungary, Glucagon-like peptide-1 analogues, Sodium-glucose co-transporter 2 inhibitors, Sulfonylureas

## Abstract

**Background:**

The prevalence of diabetes is increasing, and several new drug groups have been authorized and used successfully in the treatment of diabetes, while older drug groups are still in use. Our aim was to assess the utilization tendencies and regional differences in antidiabetic medication consumption in Hungary between 2015 and 2021 and to identify the possible determinants of regional differences in antidiabetic medication use.

**Methods:**

For this retrospective drug utilization study, yearly wholesale database was used, which provides total coverage for ambulatory antidiabetic drug sales in Hungary, including both reimbursed and non-reimbursed medications. Data were expressed as Defined Daily Dose per 1000 inhabitants per day (DDD/TID), percentage of total use and the ratio of the highest and lowest utilization values among the counties (max/min ratio). To assess the potential reasons for regional differences in antidiabetic drug use, we analyzed the associations between regional drug utilization data and possible determinants.

**Results:**

The total national antidiabetic medication use has increased by 7.6% and reached 94.8 DDD/TID in 2021. Regarding antidiabetic subgroups, the use of metformin and novel antidiabetics (DPP4Is, GLP1As and SGLT2Is) and their combinations increased in all counties, while sulfonylurea consumption decreased, and insulin use was stable. In 2021, 19.2–24.1% of the total antidiabetic medication consumption was novel antidiabetics, 39.1–47.2% metformin, 14.8–25.8% sulfonylureas and 23.6–30.5% were insulins. Regional differences in antidiabetic medication consumption were considerable mainly in the case of GLP1As (max/min ratio:3.00), sulfonylureas (2.03) and SGLT2Is (1.92) in 2021. The association between antidiabetic medication use and possible determinants was confirmed in the case of unemployment rate and sulfonylurea use, the number of public medical card holders per ten thousand inhabitants and human insulin and sulfonylurea use. GLP1As were the only antidiabetic drug group that did not correlate with any of the investigated factors.

**Conclusions:**

Although novel antidiabetic drug use was growing dynamically in Hungary, sulfonylurea use is still considerable. Differences in antidiabetic drug consumption were substantial between the regions.

**Supplementary Information:**

The online version contains supplementary material available at 10.1186/s13098-024-01334-8.

## Introduction

Diabetes is a chronic disease affecting an increasing number of people worldwide. According to the International Diabetes Federation Diabetes Atlas 2021, 537 million adults aged 20 to 79 years (10.5% of the population) had diabetes worldwide, and by 2045, this number will reach 783 million (12.2% of the expected population) [1]. In Hungary, 14.2% of adults (aged 19 and older) registered with general practitioners had diabetes in 2021, according to the database of the Hungarian Central Statistical Office [2]. The alarming rate of diabetic patients has led to intensive pharmacological research, and therapeutic approaches for diabetes have changed considerably in recent decades [3]. Novel drug groups, namely, dipeptidyl peptidase 4 inhibitors (DPP4Is), glucagon-like peptide-1 analogues (GLP1As) and sodium-glucose co-transporter 2 inhibitors (SGLT2Is), were developed and included in the therapeutic guidelines. Although metformin remains the first choice in the treatment of type 2 diabetes, GLP1As and SGLT2Is have become preferred agents in adults with type 2 diabetes with an established/high risk of atherosclerotic cardiovascular disease or chronic kidney disease [3–6]. According to the American Diabetes Association-European Association for the Study of Diabetes Consensus Report, diabetes therapy should be individualized, and clinicians should consider patient-specific factors and social determinants that affect treatment choice, such as impact on weight, cardiorenal protection, side effects (e.g., hypoglycemia), complexity of regimen, cost and availability of medication, age, education, and mental status [7]. Taking into consideration the variety of these factors, the regional differences in diabetes prevalence, and the high number of antidiabetic medications, antidiabetic therapy may vary over a wide range, both at the patient and at regional levels. These differences can be quantitative and qualitative.

Previously published data on the utilization of antidiabetic medicines in Italy and Portugal revealed substantial regional differences [8, 9]. Regional differences in medication use can be associated with several factors. Beyond the previously mentioned patient-specific factors, social determinants, disease prevalence, regional differences in health policies, differences in accessibility to healthcare and the characteristics of prescribing doctors may also influence regional differences [10]. Identifying regional differences in the utilization of medicines can be useful for developing national action plans to improve treatment strategies, optimize the allocation of healthcare resources, and consequently improve the health outcomes of diabetic patients.

Although previous studies have investigated reimbursed antidiabetic drug utilization in Hungary both at the national and patient levels, complete utilization (including both reimbursed and non-reimbursed medications) of this medication group and regional utilization differences and their possible determinants have not yet been investigated [11, 12].

Our aim was to assess the utilization tendencies and regional differences in antidiabetic medication consumption in Hungary between 2015 and 2021, and to identify the possible determinants of regional differences in antidiabetic medication use.

## Methods

For the retrospective drug utilization study, yearly wholesale data on antidiabetic drugs were kindly provided by IQVIA for each Hungarian county (19 counties and capital, covering the total Hungarian population of nearly 10 million people) for the period between 2015 and 2021. IQVIA is a multinational company, that provides clinical research services for life science research, including data on drug utilization. The database covers the total ambulatory drug sales in Hungary, including both reimbursed and non-reimbursed medications. The database contains aggregated sales data at the product level: year, region where the drug was purchased, anatomical therapeutic chemical classification code (ATC) of the active ingredient, name of the product, number of boxes and number of defined daily doses (DDDs) of each product per year per county. DDD is the average daily maintenance dose of a medication when used in adults in its main indication [13].

Data were analyzed using the WHO ATC/DDD system (version 2022), and the filtered ATC code was A10, which is drugs used in diabetes. Regional consumption data were expressed as defined daily dose per 1000 inhabitants per day (DDD/TID), and relative use was expressed as the percentage of total antidiabetic medication use [13, 14]. The following formula was used to calculate DDD/TID: (total number of DDDs used in 1 year x 1000) / (population x 365). DDD/TID is a standardized technical unit that helps to compare drug utilization across different populations [14]. When analyzing the utilization of each pharmacological subgroup, monocomponent products and combination products were added together to provide the results of the overall use of each active ingredient and each pharmacological subgroup. Consequently, the combination products were considered in both relevant pharmacological subgroups (Suppl. 2). However, when calculating the overall use of antidiabetic medication, the combination products were included only once, avoiding the addition of the same product twice.

To show the extent of regional utilization differences, the ratio of the highest and lowest utilization values among the counties was calculated (max/min ratio). To analyze time trends in the use of antidiabetic drug groups, simple linear regression was applied and described with the regression coefficient and significance (p value) of the coefficient. Statistical significance was set at *P* < 0.05. The dependent variable was the use of antidiabetic drug groups (expressed as DDD/TID), and the independent variables was time (years). The regression coefficient describes trends and shows the average annual changes, while positive coefficients indicate increasing trends, and negative coefficients indicate decreasing trends.

To assess the potential reasons for regional differences in antidiabetic drug use, we analyzed associations between regional drug utilization data for the year 2021 and possible determinants. Demographic data and possible determinants of antidiabetic drug use were extracted from the Hungarian Central Statistical Office database and the National Health Insurance Fund of Hungary report on the World Diabetes Day if relevant regional data were available [15, 16]. These extracted determinants were as follows: unemployment rate, number of public medical card holders per ten thousand inhabitants (type of financial support to reduce medical expenses for socially disadvantaged people as they can obtain specific medicine free of charge up to a monthly maximum limit), regional prevalence of diabetes, percentage of the 60 years and older among the total population, and number of attendances in diabetologic outpatient service (diabetologists) per thousand inhabitants.

Correlations were assessed using the Spearman’s rank test. Microsoft Excel (Microsoft Office, 2010, Microsoft Corp., Redmond, WA, USA), R (version 3.6.0, R Foundation for Statistical Computing Vienna, Austria) and Datawrapper (Datawrapper GmbH, Berlin, Germany) were used for data analysis and plotting.

Regarding the reimbursement system in Hungary, the National Health Insurance Fund is the sole mandatory national health insurance company. In our study, all antidiabetic active ingredients were reimbursed, but not necessarily every product. GPs can prescribe all antidiabetic medications, but in the case of insulins, GLP1As, SGLT2Is and DPP4Is for the reimbursement, regular diabetologist recommendations and follow-ups are necessary [17].

Ethical approval was not required because wholesale drug utilization data were aggregated and not linked to any patient data.

## Results

During the study period, both national and regional antidiabetic medication use showed a growing tendency, but to different extent. The total national antidiabetic medication use increased by 7.6% and reached 94.8 DDD/TID in 2021, however, in 2020, there was a peak of 97.4 DDD/TID. In most of the counties the rise in total antidiabetic consumption was considerable between 4.5% and 16.5% over the 7 years, except in the capital (Budapest) and in Győr-Moson-Sopron County (in the northwestern part of the country). The highest antidiabetic medication utilization in 2021 and the highest increase in use during the study period were observed in Békés County (in the southeastern part of the country) (Table 1). The difference in antidiabetic medication use between counties was relatively stable, the max/min ratio was between 1.37 and 1.41 during the study period and south-southwest counties tended to use more antidiabetics.

**Table 1:** National and regional trends in the use of antidiabetic drugs over the 7-year period (between 2015 and 2021)

Group name	Antidiabetics total				Insulins and combinations				Metformin and combinations				Sulfonylureas				DPP4Is and combinations				GLP1As and combinations				SGLT2Is and combinations			
	DDD/TID				DDD/TID				DDD/TID				DDD/TID				DDD/TID				DDD/TID				DDD/TID			
Region/County	2015	2021	coeff.	p	2015	2021	coeff.	p	2015	2021	coeff.	p	2015	2021	coeff.	p	2015	2021	coeff.	p	2015	2021	coeff.	p	2015	2021	coeff.	p
**Budapest**																												
Budapest	87.0	89.1	0.42	0.128	24.7	22.6	−0.32	0.089	33.9	40.4	1.14	< 0.001*	23.8	14.8	−1.46	< 0.001*	6.7	7.8	0.23	0.009*	0.9	6.3	0.86	0.001*	0.5	5.7	0.88	< 0.001*
**Pest**																												
Pest	83.4	88.5	1.09	0.018*	24.3	24.6	0.14	0.353	32.6	39.9	1.32	< 0.001*	23.3	15.2	−1.30	< 0.001*	6.0	8.0	0.40	0.001*	0.7	5.1	0.71	< 0.001*	0.2	5.2	1.20	< 0.001*
**Central Transdanubia**																												
Fejer	88.9	98.4	1.72	0.003*	24.6	24.9	0.14	0.549	36.0	46.4	1.73	< 0.001*	24.0	15.4	−1.38	< 0.001*	7.1	9.0	0.38	0.001*	0.9	5.8	0.82	< 0.001*	0.6	8.3	1.31	< 0.001*
Komarom-Esztergom	88.6	94.7	1.30	0.009*	26.0	25.2	−0.01	0.936	34.6	43.4	1.54	< 0.001*	24.8	18.4	−0.99	< 0.001*	5.5	7.0	0.29	< 0.001*	0.6	4.0	0.57	< 0.001*	0.2	5.8	1.17	< 0.001*
Veszprem	86.8	94.9	1.46	0.002*	24.6	25.2	0.14	0.427	35.8	42.5	1.15	< 0.001*	22.9	15.6	−1.18	< 0.001*	6.0	8.9	0.53	< 0.001*	0.5	5.1	0.75	< 0.001*	0.6	6.9	1.13	< 0.001*
**Western Transdanubia**																												
Gyor-Moson-Sopron	81.9	82.5	0.18	0.432	20.2	19.5	−0.07	0.504	32.5	38.8	1.07	< 0.001*	26.3	17.7	−1.42	< 0.001*	5.6	6.9	0.26	0.004*	0.6	3.3	0.44	< 0.001*	0.1	5.1	0.87	< 0.001*
Vas	98.0	102.2	0.82	0.023*	27.4	24.7	−0.41	0.065	37.8	47.7	1.68	< 0.001*	28.4	18.6	−1.60	< 0.001*	5.8	8.3	0.47	0.003*	1.1	7.0	1.00	< 0.001*	0.3	6.9	1.50	< 0.001*
Zala	96.1	111.0	2.83	< 0.001*	29.4	30.3	0.33	0.235	32.0	43.4	1.98	< 0.001*	29.6	24.2	−0.79	< 0.001*	8.6	11.8	0.60	< 0.001*	0.5	5.3	0.80	< 0.001*	0.6	8.8	1.05	< 0.001*
**Southern Transdanubia**																												
Baranya	100.9	108.9	1.48	0.006*	33.5	29.7	−0.65	0.014*	33.3	45.7	2.11	< 0.001*	29.4	21.1	−1.31	< 0.001*	7.7	9.2	0.30	0.018*	1.0	6.4	0.90	< 0.001*	0.6	8.4	1.33	< 0.001*
Somogy	97.7	109.1	2.12	< 0.001*	26.9	26.9	0.05	0.772	38.6	49.3	1.89	< 0.001*	28.2	20.5	−1.26	< 0.001*	7.4	9.1	0.34	0.003*	1.0	6.8	0.98	< 0.001*	0.5	8.9	0.83	< 0.001*
Tolna	103.1	115.5	2.30	< 0.001*	32.6	33.1	0.15	0.407	39.1	52.0	2.26	< 0.001*	25.8	17.1	−1.40	< 0.001*	8.6	11.1	0.46	0.012*	1.3	7.8	1.09	< 0.001*	0.4	9.1	0.92	< 0.001*
**Northern Hungary**																												
Borsod-Abauj-Zemplen	86.8	93.5	1.30	0.004*	26.7	26.1	−0.04	0.811	29.8	39.1	1.61	< 0.001*	26.5	20.6	−0.93	< 0.001*	5.5	8.1	0.50	< 0.001*	0.8	3.8	0.51	< 0.001*	0.7	6.9	1.05	< 0.001*
Heves	91.2	98.6	1.49	0.006*	27.2	28.5	0.32	0.231	32.1	40.6	1.50	< 0.001*	29.0	21.7	−1.11	< 0.001*	6.5	8.8	0.48	< 0.001*	0.7	3.9	0.53	< 0.001*	0.5	6.3	0.97	< 0.001*
Nograd	86.1	92.3	1.29	0.006*	23.0	23.3	0.15	0.392	30.5	38.4	1.41	< 0.001*	29.4	23.5	−0.99	< 0.001*	6.7	9.5	0.55	< 0.001*	0.6	2.6	0.32	< 0.001*	0.6	7.5	0.99	< 0.001*
**Northern Great Plain**																												
Hajdu-Bihar	81.5	87.5	1.26	0.005*	20.5	20.8	0.11	0.297	32.0	40.1	1.45	< 0.001*	26.6	19.4	−1.06	< 0.001*	6.4	8.9	0.47	< 0.001*	0.5	3.9	0.57	< 0.001*	0.3	6.8	1.08	< 0.001*
Jasz-Nagykun-Szolnok	94.3	104.5	2.16	0.003*	30.5	31.7	0.32	0.077	31.0	40.7	1.72	< 0.001*	28.2	21.9	−0.84	0.004*	7.4	10.0	0.49	< 0.001*	0.7	3.6	0.49	< 0.001*	0.3	7.1	1.08	< 0.001*
Szabolcs-Szatmar-Bereg	75.4	83.6	1.70	0.002*	25.8	25.5	0.10	0.565	25.6	35.6	1.72	< 0.001*	21.5	15.0	−0.98	< 0.001*	5.4	7.9	0.49	< 0.001*	0.6	4.0	0.57	< 0.001*	0.3	5.6	1.45	< 0.001*
**Southern Great Plain**																												
Bacs-Kiskun	90.1	99.6	1.88	< 0.001*	25.6	25.7	0.04	0.814	34.6	44.4	1.74	< 0.001*	25.8	19.6	−0.80	0.007*	8.4	10.9	0.43	< 0.001*	0.7	3.7	0.51	< 0.001*	0.5	9.1	1.49	< 0.001*
Bekes	100.2	116.7	3.24	< 0.001*	29.4	30.0	0.16	0.422	32.6	45.7	2.32	< 0.001*	33.8	30.1	−0.28	0,333	7.7	9.7	0.37	< 0.001*	0.5	3.5	0.48	< 0.001*	0.5	9.8	1.57	< 0.001*
Csongrad-Csanad	84.6	94.5	2.49	0.009*	24.6	23.4	−0.14	0.296	30.4	42.8	2.38	< 0.001*	26.4	18.7	−0.79	0,105	5.2	8.1	0.52	< 0.001*	0.7	4.4	0.60	< 0.001*	0.7	8.5	1.33	< 0.001*
**Hungary**	88.1	94.8	1.35	0.004*	25.7	25.1	−0.03	0.858	32.9	41.8	1.56	< 0.001*	25.7	18.2	−1.16	< 0.001*	6.6	8.6	0.39	0.001*	0.7	4.9	0.69	< 0.001*	0.4	6.8	1.37	< 0.001*

Table shows drug utilization data (expressed in DDD/TID) in the beginning year (2015) and in the ending year (2021). Regarding utilization trends, regression coefficient describes trends showing the average annual changes, the positive coefficient means increasing, negative coefficient means decreasing tendency

DDD/TID: Defined daily dose per 1000 inhabitants per day; coeff.: regression coefficient; GLP1A: GLP-1 analogues; DPP4I: DPP-4 inhibitors; SGLT2I: SGLT-2 inhibitors; *p < 0.05

Regarding the use of antidiabetic subgroups, large and stable interregional differences were observed. During the study period, both insulin use and interregional differences in insulin use were stable (max/min ratio: 1.65–1.70) without a clear geographical gradient (Table 1.). In 2021, insulin use was 23.6–30.5% of total antidiabetic medication consumption. In contrast to insulin utilization, metformin and sulfonylurea use showed dynamic alterations. The utilization of metformin and its combinations showed an emerging trend in all counties and reached 39.1–47.2% of the total antidiabetic medication use at the end of the study period, which means that metformin and its combinations were the most frequently used antidiabetic medications (Table 1). The south-southwest counties tended to use more metformin than the northeast counties, but the interregional differences in metformin use were the smallest among all antidiabetic drug groups (max/min ratio: 1.46 in 2021, ranging between 1.46 and 1.52). Although sulfonylurea use decreased in all counties during the study period, notable differences were observed in regional consumption (Table 1). While the use of sulfonylureas was the lowest in Budapest, with 14.8 DDD/TID in 2021, the use of this drug group was the highest in Békés County, with 30.1 DDD/TID (max/min ratio of 2.03). The relative use of sulfonylureas in different regions was still between 14.8% and 25.8% of the total antidiabetic medication consumption in 2021.

**Fig. 1:**
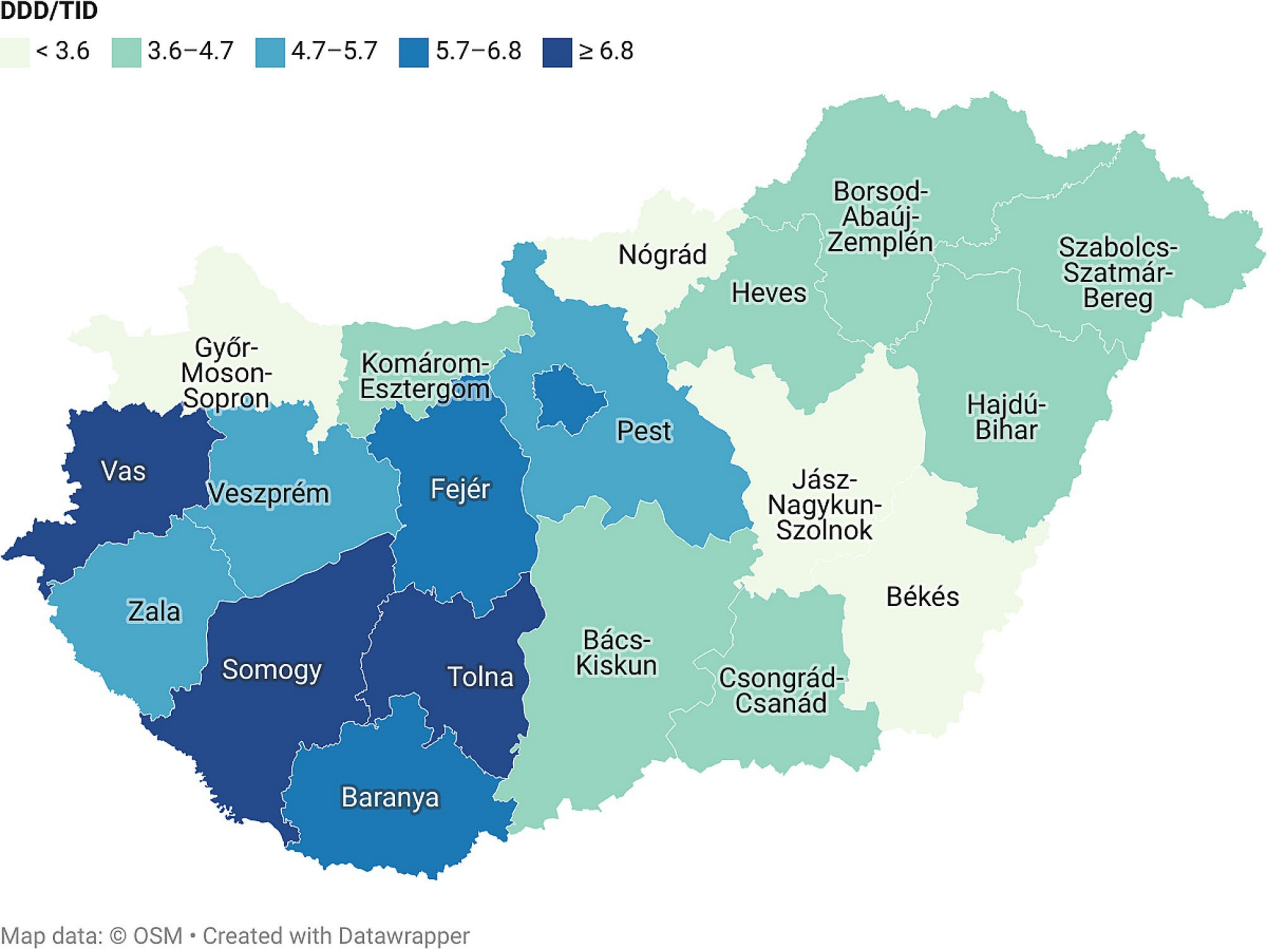
Regional differences in the use of glucagon-like peptide-1 analogues and their combinations in 2021 (expressed in DDD/TID)

The use of novel antidiabetic drug groups, namely, DPP4Is, SGLT2Is and GLP1As and their combinations, showed an emerging tendency. The use of DPP4Is was the highest among these drug groups, but after dynamic growth between 2015 and 2020, its use decreased slightly and, in some counties, SGLT2I utilization exceeded DPP4I use by 2021 (Table 1, Suppl. 1). DPP4Is, SGLT2Is, GLP1As, and their combinations accounted for 19.2–24.1% of the total use of antidiabetic drugs in Hungary in 2021. Regarding interregional differences, GLP1A use showed the highest difference among the antidiabetic drug groups in 2021 (max/min ratio: 3.00). GLP1A utilization was the highest in the western regions, mainly in the southwest (Southern Transdanubia), whereas utilization was much lower in the east, mainly in the northern regions of Hungary and the Northern Great Plain (Table 1; Fig. 1). SGLT2I use tended to be higher in the southern counties, while in the northern counties, the utilization was much lower, with a max/min ratio of 1.92 in 2021 (Table 1). In the case of DPP4Is, the difference between the regions was lower than that of the other two drug groups; the max/min ratio was 1.70 in 2021, and a clear geographical gradient was not observed.

**Fig. 2:**
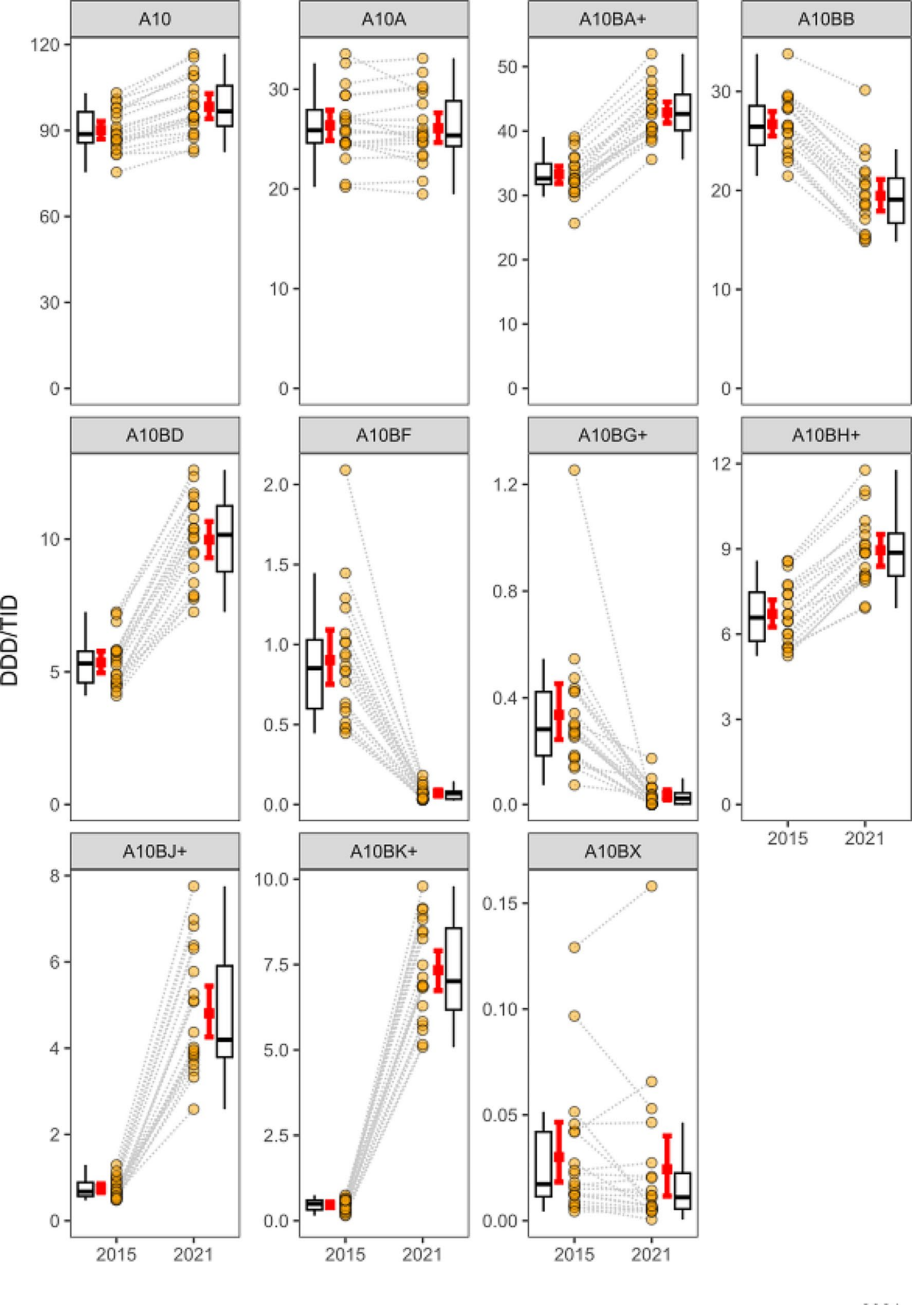
Change in regional antidiabetic drug group utilization between 2015 and 2021. Red square and lines: county average ± 95% CI; Yellow circle: county utilization data. A10: Antidiabetics total, A10A: Insulins, A10BA+: Biguanides and combinations, A10BB: Sulfonylureas, A10BD: Combinations of antidiabetics, A10BF: Alpha glucosidase inhibitors, A10BG+: Thiazolidindiones and combinations, A10BH+: dipeptidyl peptidase 4 inhibitors and combinations, A10BJ+: glucagon-like peptide-1 analogues and combinations, A10BK+: sodium-glucose co-transporter 2 inhibitors and combinations, A10BX: Glinides

The utilization of these antidiabetic subgroups expressed in DDD/TID is summarized in Table 1, and the regional utilization tendencies of different antidiabetic groups are shown in Suppl. 1. The changes in the regional differences in the utilization of antidiabetic drug groups between 2015 and 2021 are shown in Fig. 2.

The use of alpha glucosidase inhibitors, thiazolidinediones and glinides constantly decreased and were 0.07 DDD/TID, 0.04 DDD/TID and 0.03 DDD/TID in 2021, which was marginal compared to other antidiabetic subgroups. Therefore, these drug groups were not included in the tables and correlation analysis was not performed on these data.

Regarding antidiabetic medication use and possible determinants, total antidiabetic medication use and almost all investigated drug subgroups correlated positively with the percentage of those 60 years and older among the total population and the number of attendances in diabetologic outpatient services per thousand inhabitants, except for GLP1As and human insulins. The unemployment rate was correlated only with sulfonylurea use, and the number of public medical card holders per ten thousand inhabitants was correlated only with human insulin and sulfonylurea use. The regional prevalence of diabetes did not correlate with the use of any of the investigated drug groups. GLP1As were the only antidiabetic drug group that did not correlate with any of the investigated factors. The associations between antidiabetic medication use and possible determinants are presented in Table 2.

**Table 2:** Associations between antidiabetic use and possible determinants

	Antidiabetics total	Insulins	Human insulins	Analogue insulins	Metformin and combinations	Sulfonylureas	DPP4Is and combinations	GLP1As and combinations	SGLT2Is and combinations
	Correlation coefficient (p value)								
**Unemployment rate**	0.021 (0.930)	0.262 (0.265)	0.116 (0.627)	0.180 (0.446)	−0.311 (0.182)	0.468* (0.038)	0.331 (0.154)	−0.386 (0.092)	0.198 (0.402)
**Number of public medical card holders per ten thousand inhabitants**	0.203 (0.391)	0.543* (0.013)	0.463* (0.04)	0.314 (0.177)	−0.104 (0.663)	0.466* (0.038)	0.313 (0.179)	−0.260 (0.268)	0.205 (0.387)
**Regional prevalence of diabetes**	0.171 (0.470)	0.126 (0.596)	0.132 (0.578)	−0.033 (0.890)	0.304 (0.193)	−0.119 (0.618)	0.059 (0.806)	0.251 (0.286)	0.209 (0.376)
**Percentage of the 60 years and older among the total population**	0.815* (< 0.001)	0.602* (0.005)	0.173 (0.466)	0.576* (0.008)	0.553* (0.011)	0.651* (0.002)	0.741* (< 0.001)	0.177 (0.454)	0.791* (< 0.001)
**Number of attendances in diabetologic outpatient service (diabetologists) per thousand inhabitants**	0.694* (0.001)	0.670* (0.001)	0.294 (0.208)	0.568* (0.009)	0.536* (0.015)	0.479* (0.032)	0.501* (0.024)	0.174 (0.464)	0.656* (0.002)

Associations were tested with Spearman’s rank test. P values < 0.05 showed statistical significance

## Discussion

This is the first study to analyze antidiabetic drug utilization in Hungary, including not only reimbursed but also non-reimbursed antidiabetics. It is also the first study to analyze antidiabetic medication use and its determinants at the regional level in Hungary. In recent years, substantial novelties in diabetes therapy and therapeutic protocols impacted antidiabetic medication utilization patterns [11]. This retrospective drug utilization study confirmed that antidiabetic medication use has changed remarkably between 2015 and 2021 in Hungary.

In all Hungarian counties, total antidiabetic use emerged, but with an interesting peak in 2020. This utilization peak coincided with the coronavirus disease-19 outbreak when the Hungarian population tended to stock their chronic medications. This stockpiling effect was also observed in the medication utilization data of other nations [18].

Regarding antidiabetic subgroups, metformin was the most commonly used antidiabetic alone or in fixed-combination with other antidiabetic drugs (DPP4Is and SGLT2Is) during the entire study period. This was explained by the Hungarian guidelines where metformin is the first drugs of choice alone or in combination if the patients are newly diagnosed with type 2 diabetes and do not have HbA1c above 9% with catabolic symptoms [5]. The high rate of metformin use is similarly observed in Denmark, where metformin use was 39% of the total antidiabetic use in 2021 [19]. Sulfonylureas are still included in Hungarian and international therapeutic guidelines but not as preferred agents due to their side effects, such as hypoglycemia and weight gain, and furthermore, this drug group does not decrease the risk of major cardiovascular events [4, 5, 7]. Although sulfonylurea use has decreased continuously, its share of total antidiabetic medication use was still remarkable in Hungary. The utilization of sulfonylureas has shown high differences among some European countries. In Hungary the share of sulfonylurea use at the national level was 19.2% in 2021; in Denmark, it was only 3.6% in 2021, while in Romania, the sulfonylurea use was estimated to be 27.9% of the total antidiabetic medication use in 2019 [19, 20]. Despite the growing prevalence of diabetes, insulin use remained relatively stable, while the utilization of newer antidiabetics, mainly SGLT2Is and GLP1As, has emerged dynamically. The use of newer antidiabetic groups may delay the initiation of insulin therapy in type 2 diabetes, because the availability of these drug groups provides a wider choice for clinicians before considering insulin therapy [21]. Additionally, while SGLT2Is and GLP1As have positive cardiovascular and renal effects, insulin has a neutral effect in this respect, but has a high risk of hypoglycemia and weight gain [4, 5, 7]. The emerging tendency of SGLT2I and GLP1A use and the change in the use of DPP4I in 2021 is partly due to the different place of these drug groups in therapeutic guidelines. Currently, GLP1As and SGLT2Is are preferred agents in cases of established/high risk of atherosclerotic cardiovascular disease or chronic kidney disease [4, 22]. In addition, SGLT2Is are preferred in cases of established/high risk of heart failure and GLP1As are preferred if the main goal is weight management above glycemic targets [4, 22]. DPP4Is have neutral effects on weight and cardiovascular and renal problems with moderate effect on blood glucose control, and their use is preferred if the main goal is to improve glucose control without hypoglycemia in the case of elder, frail people [3, 4]. The emerging use of DPP4Is, SGLT2Is and GLP1As use has also been observed in other countries’ utilization data, such as Denmark or Portugal [9, 19].

Regarding interregional differences, we found stable and considerable variability in the use of antidiabetics which has not been previously studied. Regional differences in total antidiabetic use remained stable and low, in contrast with some antidiabetic subgroups. Insulin use and its regional differences were relatively stable in all counties, and we did not find a geographical gradient in the utilization pattern or association with regional diabetes prevalence. Although insulin use did not correlate with the unemployment rate, an association was found between insulin use and the number of public medical card holders per ten thousand inhabitants. The initiation of insulin therapy is not a financial issue, because human insulin preparations are available with 100% reimbursement (with only minimal patient co-payment of approx. 0.8 EUR/box), so the patient’s financial situation has no influence on receiving insulin therapy [17]. However, if patients with type 2 diabetes need to receive insulin analogues that are more expensive, these preparations are available with 50% or 100% reimbursement coverage depending on the patients’ HbA1c levels [17, 23]. Therefore, patients with a poorer financial situation are more likely to receive human insulin therapy than the more expensive insulin analogues. This is supported by the positive association between human insulin use and the number of public medical card holders. The positive correlation between insulin use and the number of attendances in diabetologic outpatient service per thousand inhabitants may be explained by the fact that insulin can be prescribed with reimbursement only under regular diabetologist supervision [17]. The positive correlation between insulin use and percentage of people with age 60 years or older can be explained by the fact that older people are more likely to have diabetes for a longer period of time, therefore, their diabetes is more likely to have progressed to the stage where insulin therapy is necessary to be initiated.

In the case of metformin, the relatively low difference in use among counties and the lack of associations with socioeconomic factors may be explained by the high use of metformin in all counties because of therapeutic recommendations, and its affordability and availability. Metformin alone is relatively inexpensive and therefore does not impose a high financial burden on patients, and GPs can prescribe these agents without regular supervision by diabetologists [17]. Other fixed-dose preparations, mainly with DPP4Is and SGLT2Is are available with 70% reimbursement, but only under regular diabetologist supervision [17].

In addition to the considerable utilization of sulphonylureas, we found high differences among counties. The high use of sulfonylureas in Hungary can be explained by some factors. Sulfonylureas are inexpensive agents, especially in contrast to newer drug groups, such as GLP1As, which are the most expensive antidiabetic drug group. In Hungary, the full price of sulfonylurea is approximately 0.06–0.14 EUR/DDD and available with 55% reimbursement. Although GLP1As are available with 70% reimbursement coverage, their full price is much higher, approximately 2.12–4.72 EUR/DDD. Additionally, sulfonylureas, similar to metformin, are easily accessible because GPs can prescribe these drugs, while other drug groups (e.g. novel antidiabetic drug groups) can be prescribed with reimbursement only under regular diabetologists’ supervision [17]. This seemed to be confirmed by the positive associations with some socioeconomic factors such as unemployment rate, the number of public medical card holders per ten thousand inhabitants and percentage of the 60 years and older among the total population.

Regarding DPP4I use, although the max/min difference between regions was 1.70 in 2021, a notable regional pattern and association with socioeconomic factors could not be detected. Higher interregional differences were found in the case of SGLT2Is, and higher use was observed in the southern counties; however, we did not find any relevant socioeconomic factors that explains these differences. The utilization of GLP1As showed the largest interregional differences among antidiabetics (max/min ratio 3.00 in 2021). We did not find any socioeconomic factors that explained the detected southwest-northeast gradient. Although we did not detect an association with socioeconomic factors, many issues may influence the use of these drug groups. First, their price (mainly GLP1As and SGLT2Is) was significantly higher than that of metformin or SU. Second, these drugs can only be prescribed with reimbursement by GPs under the recommendations of diabetologists [17], which may complicate access to these medications for some patients. Additionally, most GLP1As are subcutaneous injections, which may be difficult for some patients to accept, although one orally administered GLP1A has been available since 2020.

Our data clearly show that drug choice depends not only on socioeconomic factors, but also on numerous other factors, which may be difficult to detect at the population level, as the choice of drug is highly individualized. We did not find any other studies that investigated the possible determinants of regional antidiabetic medication use. However, in one study that investigated geographical variation in antibiotic use and its possible determinants in Hungary, large interregional differences and associations with some socioeconomic factors were found [24].

Comparing the results of our study, which included both reimbursed and non-reimbursed medication use, to the results of a previously published study based on only reimbursed medication use, it was revealed that there were considerable differences in the results [11]. The overall use of antidiabetic medications was 24% higher in our study in 2015 compared to the use of only reimbursed medication. In the cases of most antidiabetic subgroups, the differences were very small (the lowest was for insulins being only 1.2%) but was enormous in the case of metformin. The overall use of metformin in our study was more than double that of the reimbursed only metformin use. This can be explained by the fact that while most antidiabetic medications are reimbursed, some widely used metformin products are not.

The present study has some strengths and limitations that must be considered. As far the strengths, first, to the best of our knowledge this is the first study to investigate both total national and interregional antidiabetic medication utilization trends and differences in Hungary. Second, the database covers total antidiabetic drug sales in Hungary, including both reimbursed and non-reimbursed medications, and our study has total population coverage (nearly 10 million people), which enables us to detect a complete and detailed picture of the national and interregional trends and differences in antidiabetic medication use.

Regarding the limitations of this retrospective study, a wholesale database containing antidiabetic medication sales for pharmacies was used. Due to the nature of the data source, it provides a slight overestimation of antidiabetic use, as not all the medications acquired by pharmacies reach the patients for various reasons (e.g. medication expires before selling, damaged medications). In addition, the data were aggregated and it was not possible to distinguish between the drug claims of patients with type 1 and type 2 diabetes. Some antidiabetics, such as metformin, may be used for indications other than diabetes, but our data did not contain information about indications of use. The database contains sales data of reimbursed and non-reimbursed medicines, however, the differentiation among these drug categories was not possible in the present study. In some cases, data on regional level were not available for potentially relevant determining factors. It should be noted that this study aimed to analyze antidiabetic medication use at the population level and explore the changes over time, but did not aim to evaluate the appropriateness of the choice of treatment, as it could only be performed on individual patient-level medication use and clinical data.

## Conclusion

In this study, we provided a detailed picture of antidiabetic medication use patterns in Hungary at both the national and regional levels. Although DPP4I, GLP1A and SGLT2I use was dynamically growing in Hungary, the share of sulfonylurea use is still considerable. Differences in antidiabetic drug consumption are substantial between regions, mainly in the case of GLP1As, SGLT2Is and sulfonylureas. The association between socioeconomic factors and regional drug use was confirmed only for sulfonylureas. The choice of therapy is highly individual and may depend on several patient- and healthcare-related factors; therefore, population level determining factors cannot necessarily explain regional differences. Future analysis of patient level data may help identify patient related and healthcare related factors that possibly contribute to regional differences in antidiabetic medication use.

### Electronic supplementary material

Below is the link to the electronic supplementary material.

**Supplementary Material 1:** Utilization tendencies of antidiabetic medication in Hungarian regions between 2015 and 2021.

**Supplementary Material 2:** Available fixed-dose combinations in Hungary during the study period

**Supplementary Material 3:** Calculation of DDD/TID

## Data Availability

No datasets were generated or analysed during the current study.

## Abbreviations

DPP4I: Dipeptidyl peptidase 4 inhibitor
GLP1A: Glucagon-like peptide-1 analogue
SGLT2I: Sodium-glucose co-transporter 2 inhibitor
ATC: Anatomic therapeutic chemical classification code
DDD: Defined daily doses
DDD/TID: Defined daily dose per 1000 inhabitants per day
HbA1c: Glycated hemoglobin A1c
