# Supplementary material for: Trends and regional differences in antidiabetic medication use: a nationwide retrospective observational study
Source: Diabetol Metab Syndr. 2024 Apr 24;16:88. doi: 10.1186/s13098-024-01334-8 (PMC11044416; doi:10.1186/s13098-024-01334-8)
Supplement: Supplementary file 1 — Supplementary Material 1: Utilization tendencies of antidiabetic medication in Hungarian regions between 2015 and 2021. [file 13098_2024_1334_MOESM1_ESM.pdf]

Supplementary material 1.: Utilization tendencies of antidiabetic medication in Hungarian regions between 2015 and 2021

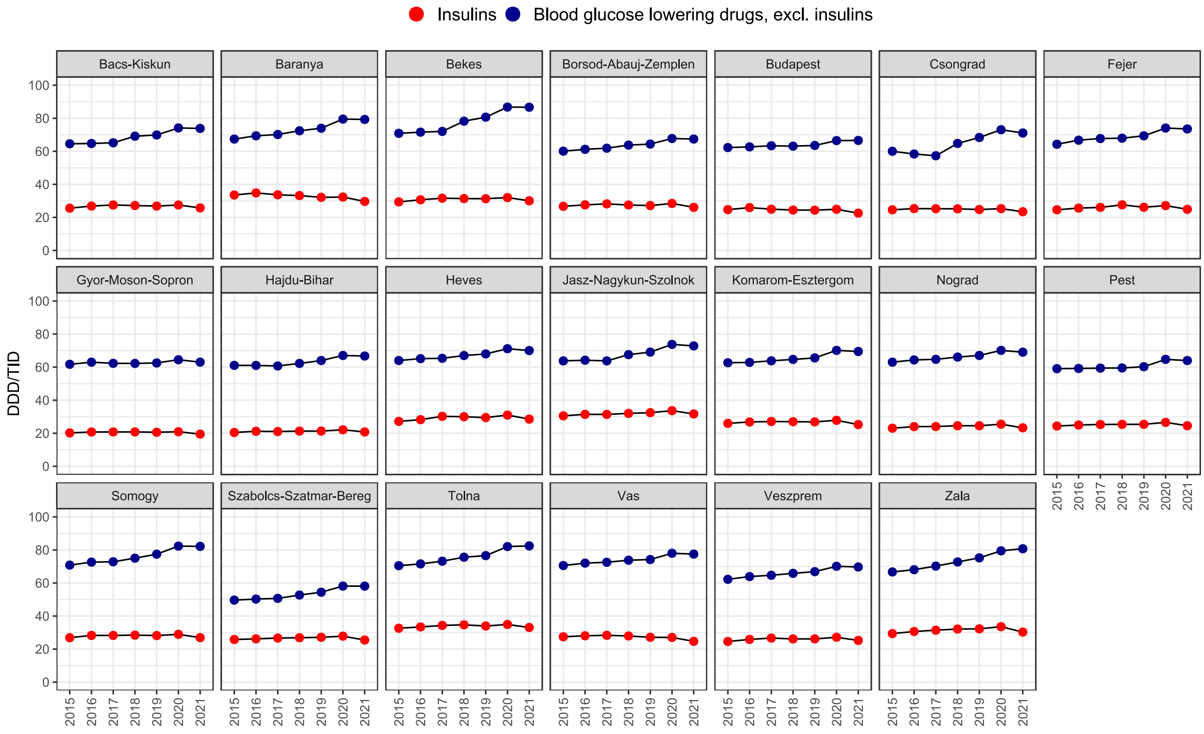

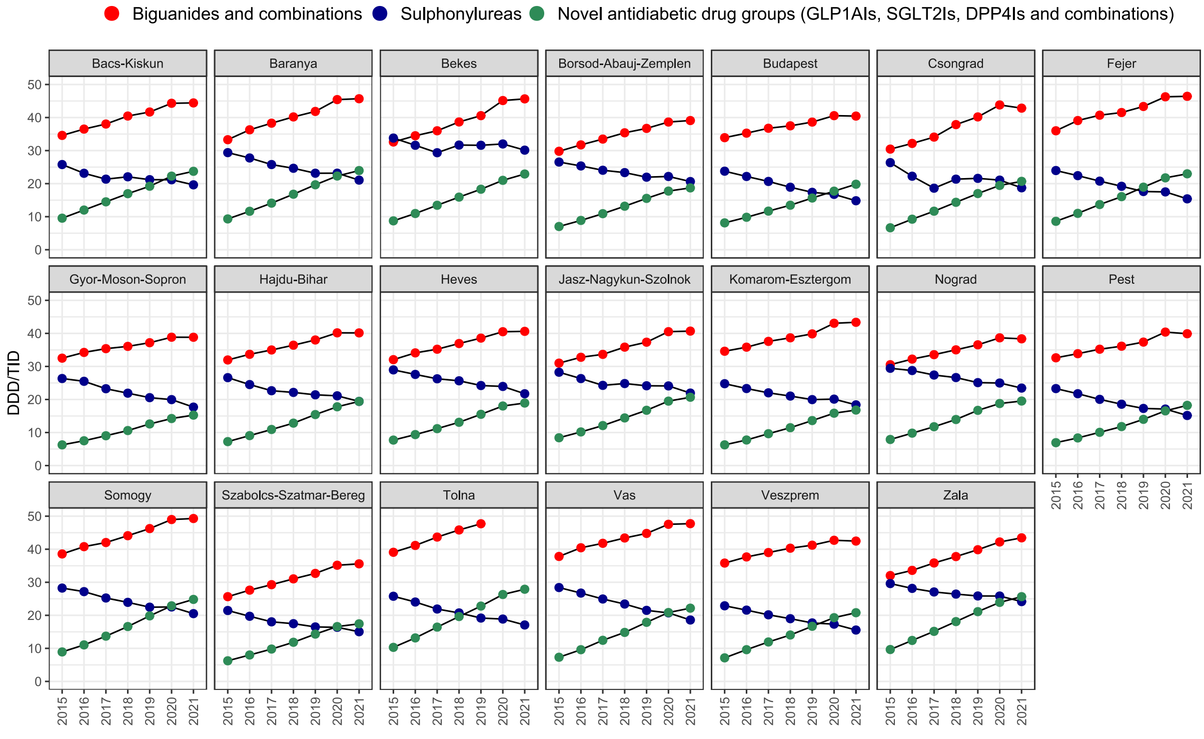

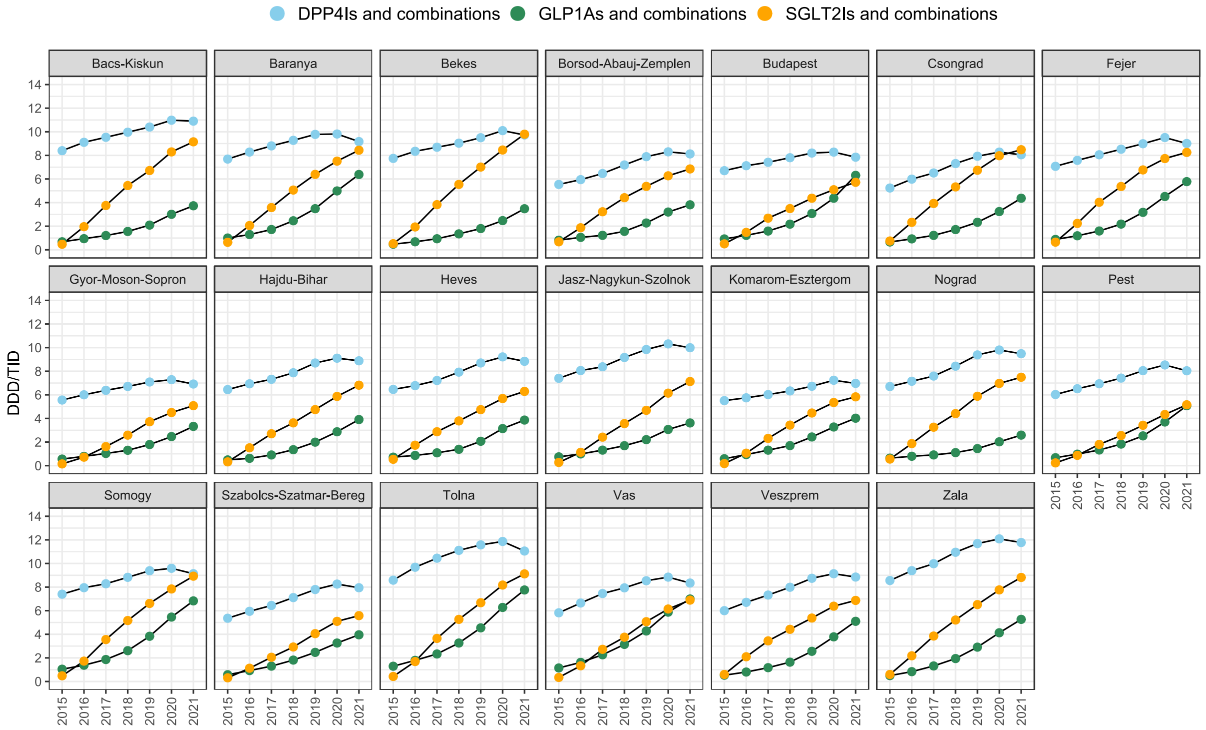
DPP4Is and combinations
GLP1As and combinations
SGLT2Is and combinations
DDD/TID
Bacs-Kiskun
Baranya
Bekes
Borsod-Abauj-Zemplen
Budapest
Csongrad
Fejer
Gyor-Moson-Sopron
Hajdu-Bihar
Heves
Jasz-Nagykun-Szolnok
Komarom-Esztergom
Nograd
Pest
Somogy
Szabolcs-Szatmar-Bereg
Tolna
Vas
Veszprem
Zala
