# Supplementary material for: Trends and regional differences in antidiabetic medication use: a nationwide retrospective observational study
Source: Diabetol Metab Syndr. 2024 Apr 24;16:88. doi: 10.1186/s13098-024-01334-8 (PMC11044416; doi:10.1186/s13098-024-01334-8)
Supplement: Supplementary file 2 — Supplementary Material 2: Available fixed-dose combinations in Hungary during the study period [file 13098_2024_1334_MOESM2_ESM.pdf]

Supplementary material 2.: Available fixed-dose combinations in Hungary during the study period

Pharmacological class:	Insulin and combinations	Metformin combinations	DPP4Is combinations	GLP1As combinations	SGLT2Is combinations
**Name of active ingredients:**	Insulin glargine and lixisenatid<br>Insulin degludec and liraglutide	Metfromin and rosiglitazone<br>Metformin and pioglitazone<br>Metfromin and sitagliptin<br>Metfromin and vildagliptin<br>Metformin and saxagliptin<br>Metformin and linagliptin<br>Metformin and alogliptin<br>Metfromin and dapagliflozin<br>Metfromin and empagliflozin<br>Metformin and ertugliflozin	Metfromin and sitagliptin<br>Metfromin and vildagliptin<br>Pioglitazone and alogliptin<br>Metformin and saxagliptin<br>Metformin and linagliptin<br>Metformin and alogliptin<br>Sitagliptin and ertugliflozin	Insulin glargine and lixisenatid<br>Insulin degludec and liraglutide	Metfromin and dapagliflozin<br>Metfromin and empagliflozin<br>Metformin and ertugliflozin<br>Sitagliptin and ertugliflozin

Supplemental reference:

1. National Health Insurance Fund of Hungary. Public Medication Database. Available at

https://www.neak.gov.hu/felso_menu/szakmai_oldalak/gyogyszer_segedeszkoz_gyogyfurdo_tamogatas/egeszsegugyi_vallalkozasoknak/pupha/Vegleges_PUPHA. Last accessed 04 March 2024.
