# Supplementary material for: Trends and regional differences in antidiabetic medication use: a nationwide retrospective observational study
Source: Diabetol Metab Syndr. 2024 Apr 24;16:88. doi: 10.1186/s13098-024-01334-8 (PMC11044416; doi:10.1186/s13098-024-01334-8)
Supplement: Supplementary file 3 — Supplementary Material 3: Calculation of DDD/TID [file 13098_2024_1334_MOESM3_ESM.pdf]

Supplementary material 3.: Calculation of DDD/TID

Example: Total national antidiabetic utilization in 2015

The sum of the DDDs of antidiabetic medications in 2015 in Hungary from the IQVIA database: 316 674 671,7

The population in Hungary in 2015: 9 843 028

The following formula was used to calculate DDD/TID: (total number of DDDs used in 1 year x 1000) / (population x 365).

Defined Daily Dose per 1000 inhabitants per day = (316 674 671,7x1000) / (9 843 028x365)= 88.1 DDD/TID
